# Synthesis and electrical characterization of rhenium-doped WS_2_ nanotubes

**DOI:** 10.1039/d6na00219f

**Published:** 2026-05-29

**Authors:** Abdul Ahad, Shigeki Saito, Ryuji Higashinaka, Ken Sakayauchi, Masaharu Kikuchi, Satoshi Kusaba, Zheng Liu, Yasushi Hirose, Kazuhiro Yanagi

**Affiliations:** a Department of Physics, Tokyo Metropolitan University Hachioji Tokyo 192-0397 Japan yanagi-kazuhiro@tmu.ac.jp; b Department of Physics, Comilla University Cumilla-3506 Bangladesh; c Department of Chemistry, Tokyo Metropolitan University Hachioji Tokyo 192-0397 Japan; d Muti-Material Research Institute, National Institute of Advanced Industrial Science and Technology (AIST) Nagoya 463-8560 Japan

## Abstract

Tungsten disulfide nanotubes (WS_2_-NTs) have attracted significant interest as one-dimensional semiconducting materials for electronic and optoelectronic devices. The development of controlled doping techniques is essential for tuning their electronic properties and enhancing device performance. In this study, we performed rhenium substitution in WS_2_-NTs with diameters of approximately 10 nm *via* the chemical vapor transport method. The concentration of substituted Re was estimated to be approximately 1.0 at%. Structural incorporation of Re atoms into the WS_2_-NT lattice effectively modified the electrical properties. As a result, the Re-doped WS_2_-NTs exhibited electrical conductivity almost three orders of magnitude higher than that of pristine WS_2_-NTs. These findings reveal a strong correlation between heteroatom-induced structural modification and electrical performance, demonstrating the potential of Re doping for tailoring WS_2_-NTs towards advanced nanoelectronic applications.

## Abstract 2

Tungsten disulfide nanotubes (WS_2_-NTs) have attracted significant interest as one-dimensional semiconducting materials. Here, we demonstrate a rhenium substitution technique for WS_2_-NTs that enhances their electrical conductivity.

## Graphical abstract

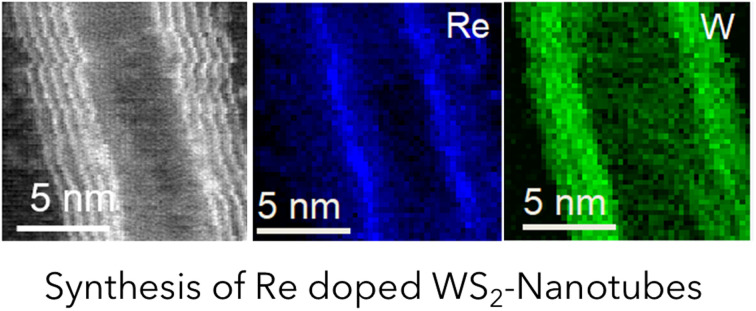

## 1. Introduction

Nanodevices utilizing one-dimensional (1D) semiconducting nanomaterials have attracted considerable attention for advanced electronic and optoelectronic applications, including field-effect transistors,^1^ photodetectors,^2^ nonlinear optical devices,^3^ and nanoscale light emitters.^4^ Miniaturized semiconductor devices based on nanostructured materials are expected to exhibit faster carrier transport, higher efficiency, and lower energy consumption.^5^

Tungsten disulfide nanotubes (WS_2_-NTs), a subclass of transition metal dichalcogenide (TMDC) NTs, are promising semiconducting nanomaterials. WS_2_-NTs possess chiral layered tubular structures and exhibit semiconducting behavior regardless of chirality.^6^ Owing to their layered structure, WS_2_-NTs have fewer surface defects and dangling bonds than other 1D semiconductor nanomaterials, such as silicon nanowires.^7^ Furthermore, unlike carbon NTs, WS_2_-NTs do not contain metallic species originating from chirality-dependent electronic structures, which is advantageous for semiconductor device applications.

For practical semiconducting applications, the development of controllable doping techniques is essential because doping plays a critical role in device architectures such as p–n junctions, field-effect transistors, logic circuits, and integrated electronic systems.^8,9^ Regarding two-dimensional TMDC sheets, various doping strategies have been extensively investigated, including surface adsorption of dopant molecules,^10^ substitutional doping *via* chemical exchange reactions,^11^ defect-induced doping,^12^ direct vapor transport,^13,14^ and plasma or ion treatment.^15^ However, a few studies have reported doping techniques for WS_2_-NTs.^16,17^ Moreover, doping has thus far only been demonstrated for TMDC-NTs with relatively large diameters (50–100 nm).^16^

Recently, several groups have successfully synthesized WS_2_-NTs with diameters as small as 10 nm.^18–20^ In such small-diameter NTs, strain effects significantly influence the electronic structure, including band-gap narrowing, leading to properties distinct from those of larger-diameter NTs (50–100 nm).^18,21^ These findings raise the important question of whether conventional doping techniques can be directly applied to small-diameter WS_2_-NTs. The applicability and effectiveness of substitutional doping in highly curved small-diameter WS_2_-NTs remain largely unexplored. Thus, an investigation of doping methods for such highly curved NTs is required.

In this study, we investigated rhenium doping into ∼10 nm-diameter WS_2_-NTs. Re doping has been widely investigated for two-dimensional TMDC flakes and sheets.^22–28^ Although Re doping has also been reported for WS_2_-NTs with relatively large diameters,^16,17^ the reported doping concentrations were limited to 0.07–0.5 at%.^16^ It is known that Re doping can enhance the electrical conductivity of TMDC materials^23–25^ and induce the metallic 1T phase in WS_2_-NTs,^17^ suggesting that Re incorporation can strongly modify the electrical properties of WS_2_-NTs. In this study, a chemical vapor transport (CVT) method was employed for Re doping into small-diameter WS_2_-NTs, and Re doping was successfully achieved. The concentration of substituted Re was estimated to be approximately 1.0 at%, and the Re-doped WS_2_-NTs exhibited electrical conductivity almost three orders of magnitude higher than that of pristine WS_2_-NTs.

## 2. Materials and methods

### 2.1 Synthesis of Re-doped WS_2_-NTs

**Fig. 1:**
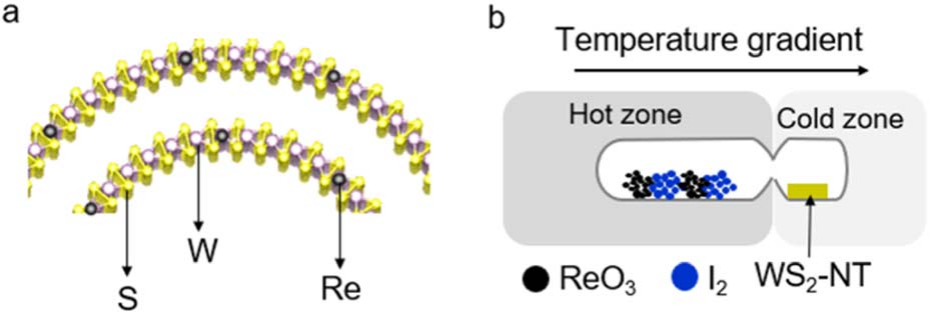
(a) Schematic illustration of Re atom substitution concept into the WS_2_-NT lattice. (b) Synthesis procedure of Re-doped WS_2_-NTs *via* the chemical vapor transport (CVT) method. In this process, I_2_ and ReO_3_ powders were placed in the high-temperature region, while pristine WS_2_-NTs were positioned in the low-temperature region. The reaction was conducted inside a high-vacuum sealed quartz ampoule.

The synthesis scheme for Re-doped small-diameter WS_2_-NTs is illustrated in Fig. 1b. First, pristine WS_2_-NTs with diameters of 10–20 nm were synthesized using our previously reported chemical vapor deposition (CVD) method.^19^ Subsequently, Re atoms were incorporated into the WS_2_-NT lattice *via* a chemical vapor transport (CVT) process.

To synthesize pristine WS_2_-NTs, W_18_O_49_ nanowires (NWs) (Fig. S2) were first grown on *c*-plane sapphire substrates by a CVD process using high-purity WO_2.9_ powder (99.99%, Thermo Scientific) as the precursor. The obtained NWs were subsequently sulfurized using sulfur lumps (99.99%, Tokyo Chemical Industry Co., Ltd) to form pristine WS_2_-NTs (Fig. S3).

For Re substitution into the WS_2_-NT lattice, a CVT process was conducted in a sealed quartz ampoule under high vacuum (approximately 5 × 10^−5^ to 10^−4^ Pa) conditions (See SI section 1.1 and Fig. 1b). The ampoule was then placed in a two-zone electric furnace. ReO_3_ powder (KOCH Chemicals Ltd) and iodine (I_2_, 99.99%, Sigma-Aldrich), used as the Re precursor and transport agent, respectively, were placed in the high-temperature zone, while the pristine WS_2_-NTs on sapphire substrates were positioned in the low-temperature zone. The temperature gradient between the two zones promoted the transport of Re species towards the WS_2_-NTs, enabling substitutional doping. The key synthesis parameters, including the temperature gradient (Fig. S4), reaction time (Fig. S5), and amounts of ReO_3_ and I_2_ (Fig. S6), were systematically optimized.

The detailed optimization procedures are described in the SI. The optimized conditions for Re-doped WS_2_-NT synthesis were as follows: a temperature gradient of 700–500 °C, a reaction time of 10 h, 12 mg of ReO_3_, and 15 mg of I_2_ for one sapphire substrate on which pristine WS_2_-NTs were synthesized by the above-described method (Fig. S1).

### 2.2 Characterizations

The morphologies of the CVD-grown W_18_O_49_ NWs, pristine WS_2_-NTs, and Re-doped WS_2_-NTs were characterized using scanning electron microscopy (SEM; Phenom ProX, Thermo Fisher Scientific Inc.) and field-emission SEM (FESEM; JSM-7800F PRIME and JSM-7100F, JEOL Ltd). Transmission electron microscopy (TEM) was performed using JEM-3200FS, JEM-2100F, and JEM-2010F microscopes (JEOL Ltd). Scanning TEM (STEM) and energy-dispersive X-ray spectroscopy (EDS) analyses were conducted using a JEM-ARM200F microscope (JEOL Ltd).

The diameter distributions and interlayer spacings of the NTs, as well as the lattice fringes of the NWs, were analyzed from the TEM images using Gatan DigitalMicrograph software. Annular bright-field (ABF)-STEM imaging was performed using a JEM-ARM200F ACCELARM microscope equipped with a cold field-emission gun and double CEOS spherical aberration correctors, operated at 120 kV. Electron energy-loss spectroscopy (EELS) elemental mapping was performed using a GIF Continuum system attached to the JEM-ARM200F operated at 200 kV.

Raman spectra were acquired using a WITec Alpha300 RAS system with a 532 nm excitation laser. The device structures and NT diameters were further characterized by atomic force microscopy (AFM; Bruker).

For electrical measurements, pristine and Re-doped WS_2_-NTs were transferred onto Si/SiO_2_ substrates with a 300 nm SiO_2_ layer. Two-terminal electrodes (Ti/Au = 10 nm/100 nm) were fabricated on individual ropes of pristine and Re-doped WS_2_-NTs using a standard photolithography process. The channel length was 3 µm. Electrical measurements were conducted under ambient conditions at room temperature.

## 3. Results and discussion

### 3.1 Structural evaluation

First, the structure of the pristine WS_2_-NTs used in this study was examined. The FESEM image (Fig. S3a) shows that the pristine WS_2_-NTs were densely distributed and free from flake-like byproducts. The high-resolution TEM (HRTEM) image (Fig. S3b) revealed well-defined crystalline NT walls with interlayer spacings ranging from 0.62 to 0.65 nm, consistent with the layered structure of 2H-WS_2_-NTs. The average diameter of the WS_2_-NTs was 13.24 ± 5.08 nm (Fig. S3c).

The Raman spectra of the pristine WS_2_-NTs (Fig. S3d) exhibited intense peaks at 354 and 419 cm^−1^, corresponding to the *E*^1^_2g_ and *A*_1g_ vibrational modes of 2H-WS_2_, respectively.^20,29^ These results confirm the successful synthesis of high-quality pristine WS_2_-NTs.

**Fig. 2:**
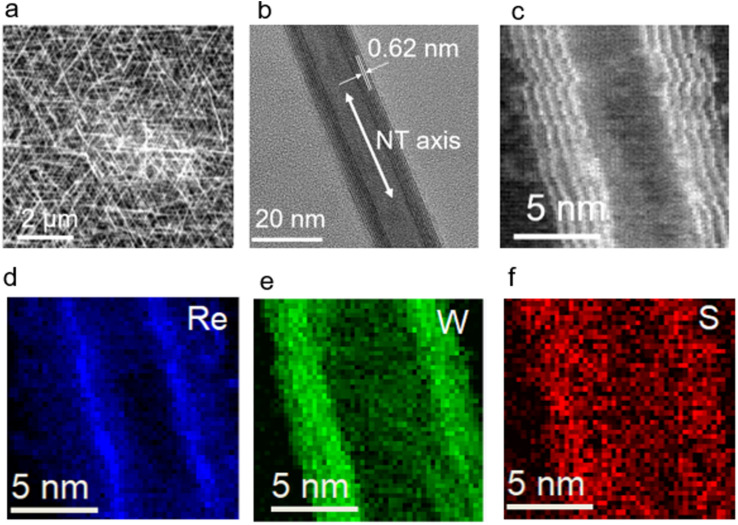
(a) SEM, (b) HRTEM, and (c) HAADF-STEM images of Re-doped WS_2_-NTs. The interplanar spacing varied from 0.62–0.65 nm. (d–f) EELS elemental maps of Re-doped WS_2_-NTs. The mapping revealed a higher concentration of Re atoms at the NT walls than within the hollow interior (d), suggesting that Re was incorporated within the layers of the WS_2_-NTs.

The structures of the Re-doped WS_2_-NTs were subsequently investigated. Fig. 2a and b show SEM and HRTEM images of the Re-doped WS_2_-NTs, respectively. The NTs retained morphologies and crystalline structures comparable to those of the pristine WS_2_-NTs (Fig. S3a and b), indicating that the CVT doping process did not significantly damage the NT structure.

Direct identification of Re atoms within the WS_2_-NT lattice was challenging because of the relatively low doping concentration. In addition, Re atoms are expected to induce only minimal lattice distortion. Furthermore, the atomic number of Re is close to that of W, resulting in only weak contrast differences in the high-angle annular dark-field (HAADF)-STEM images (Fig. 2c). Nevertheless, electron energy-loss spectroscopy (EELS) mapping (Fig. 2d–f) revealed a higher Re concentration at the NT walls than in the hollow interior region (Fig. 2d), suggesting that Re atoms were incorporated into the WS_2_-NT layers.

### 3.2 Elemental analysis

**Fig. 3:**
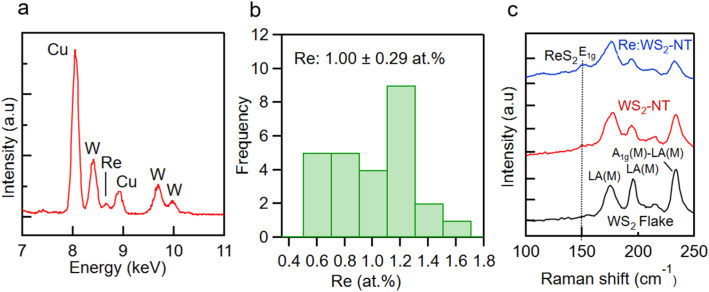
(a) EDS spectrum of Re-doped WS_2_-NTs. (b) Histogram of Re concentrations, summarizing the EDS analysis performed on multiple NTs. (c) Raman spectra of WS_2_ flake, pristine, and Re-doped WS_2_-NTs around 150 cm^−1^.

The EDS spectra of the Re-doped WS_2_-NTs indicated that distinguishing W and Re signals is difficult in the M-line region because of the small energy separation and substantial overlap of their characteristic X-ray emission peaks (Fig. S7). However, distinct W and Re peaks were clearly identified in the l-line region (Fig. 3a), enabling reliable elemental analysis.

EDS measurements were performed on multiple samples and individual NTs. A histogram of Re concentrations is shown in Fig. 3b, demonstrating an average Re concentration of approximately 1.00 ± 0.29 at%. These results indicate relatively uniform Re incorporation among the examined NTs.

At such doping concentrations, the possibility of Re agglomeration should be considered carefully. However, no evidence of significant Re aggregation was observed. The HRTEM image (Fig. 2b) shows highly crystalline NT walls without detectable amorphous layers. In addition, the EELS mapping results (Fig. 2d–f) support the homogeneous incorporation of Re within the NT walls, rather than the formation of isolated Re-rich clusters. If substantial Re agglomeration had occurred, localized high-intensity Re domains would be expected in the EELS maps. Therefore, these observations suggest that Re atoms are incorporated into the WS_2_-NT lattice without significant agglomeration.

To further confirm Re incorporation into the WS_2_-NTs, the Raman spectra of WS_2_ flakes, pristine WS_2_-NTs, and Re-doped WS_2_-NTs were measured (Fig. 3c). Several low-frequency Raman modes characteristic of WS_2_ were observed, consistent with previous reports.^30,31^ Because the Re concentration was relatively low, Raman features associated with ReS_2_ were expected to be weak. Nevertheless, the Re-doped WS_2_-NTs exhibited an additional weak peak at approximately 150 cm^−1^, corresponding to the in-plane *E*_g_ vibrational mode of ReS_2_.^32^ These results provide additional evidence for successful Re incorporation into the WS_2_-NT lattice. These structural and spectroscopic analyses collectively demonstrate successful Re incorporation into small-diameter WS_2_-NTs while preserving the crystalline NT structure.

### 3.3 Electrical transport properties

**Fig. 4:**
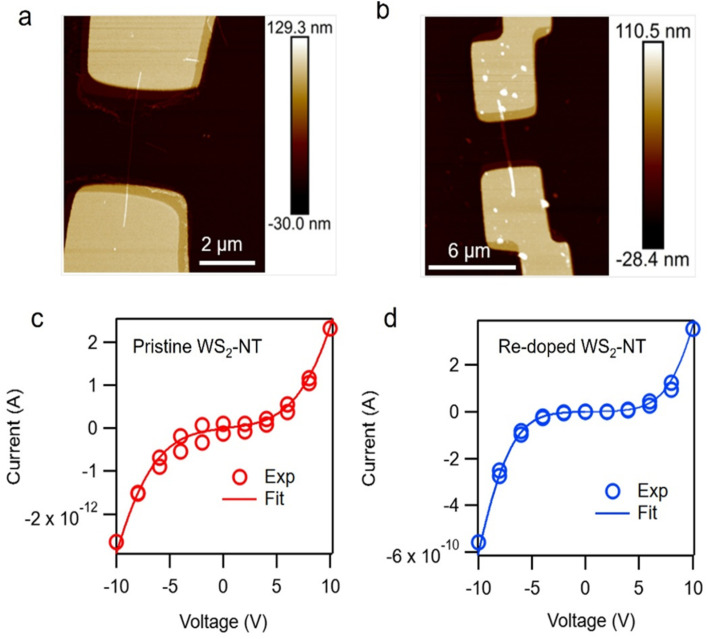
AFM images of the fabricated device structures using pristine (a) and Re-doped WS_2_-NTs (b), with outer diameters of 19.4 nm and 11.3 nm, respectively. The channel lengths are both 3.0 µm. Current voltage (*I*–*V*) characteristics of the pristine (c) and Re-doped WS_2_-NTs (d) measured at room temperature. Both curves exhibit nonlinear behaviour, indicating a Schottky barrier at the interface between the Ti/Au electrodes and NTs. Experimental data (Exp) are solid circles, and fitting lines (Fit) based on a M–S–M model are solid lines.

Fig. 4a and b show AFM images of the fabricated device structures used for electrical characterization of pristine and Re-doped WS_2_-NTs, respectively. Typical current–voltage (*I*–*V*) characteristics of the pristine and Re-doped WS_2_-NT devices are presented in Fig. 4c and d. Both devices exhibited nonlinear *I*–*V* behavior, which is commonly observed in metal–semiconductor–metal (M–S–M) systems, owing to the formation of Schottky barriers at the metal/semiconductor interfaces.^33–35^

Based on the standard analytical procedure for the M–S–M model,^21^ the electrical conductivities of pristine and Re-doped WS_2_-NTs were evaluated (details are provided in SI section 2). The electrical conductivity of pristine WS_2_-NTs was estimated to be 2.3 × 10^−2^ S m^−1^, whereas that of the Re-doped WS_2_-NTs reached 1.1 × 10^1^ S m^−1^, corresponding to an enhancement of almost three orders of magnitude after Re doping.

This substantial conductivity enhancement is consistent with previous reports showing that Re incorporation increases the electrical conductivity of TMDC sheets,^23–25^ NTs, and fullerene-like nanoparticles.^16^ Carrier densities (Table 1) were extracted from logarithmic analyses of the *I*–*V* curves (Fig. S8a and b), while the Schottky barrier heights (Table 1) were estimated by fitting the nonlinear transport behavior using the M–S–M model.

**Table 1:** Results obtained from fitting experimental *I*–*V* curves of pristine and Re-doped WS_2_-NT devices

Parameters	Value	
	Pristine WS_2_-NT	Re-doped WS_2_-NT
Diameter, *D* (nm)	19.4	11.3
Schottky barrier height, *Φ*_b_ (eV)	0.75	0.67
Conductivity, *σ* (S m^−1^)	2.3 × 10^−2^	1.1 × 10
Carrier density, *n* (cm^−3^)	3.0 × 10^17^	4.9 × 10^17^

The extracted carrier density increased moderately after Re doping. Previous studies have suggested that Re substitution shifts the chemical potential towards the conduction band,^32,33^ which can contribute to the observed increase in carrier density. However, the relatively small increase in carrier density alone may not fully account for the almost three-orders-of-magnitude enhancement in electrical conductivity.

Previous studies have also reported that Re incorporation can induce the formation of the metallic 1T phase in WS_2_-based nanostructures.^17^ The presence of such a metallic phase could additionally contribute to the enhanced electrical conductivity observed in Re-doped WS_2_-NTs. Although direct evidence of the 1T phase was not obtained in the present study, partial formation of the 1T phase is one possible reason for the significant conductivity enhancement.

Overall, these results demonstrate that Re incorporation strongly modifies the electrical transport properties of small-diameter WS_2_-NTs.

## 4. Conclusions

In this study, we demonstrated Re doping of small-diameter WS_2_-NTs *via* the CVT method. Effective incorporation of Re into the WS_2_-NT lattice was achieved, with an estimated Re concentration of approximately 1.0 at%. The structural incorporation of Re significantly modified the electrical transport properties of the WS_2_-NTs. As a result, the Re-doped WS_2_-NTs exhibited electrical conductivity almost three orders of magnitude higher than that of pristine WS_2_-NTs. However, since the electrical measurements were performed using a two-terminal configuration, four-terminal measurements will be necessary for a more detailed understanding of the intrinsic electrical properties. The CVT technique is not readily scalable, and thus alternative doping methods should be explored for future nanoelectronic applications of WS_2_-NTs. The findings of this study demonstrate that heteroatom incorporation is an effective strategy for tuning the electrical properties of small-diameter WS_2_-NTs. These results further highlight the potential of Re-doped WS_2_-NTs for future nanoelectronic applications.

## Author contributions

K. Y. proposed the project and designed the work. A. A. and R. H. performed the synthesis of Re-doped WS_2_-NTs. A. A. conducted FE-SEM, EDS, and TEM measurements. A. A, K. Y. and S. K. discussed the properties. A. A., M. K., S. S. and K. S. fabricated the nanodevices and measured the AFM and analysed the electrical properties. Z. L. contributed to the STEM and EELS measurements. Y. H. supported the FE-SEM measurements. All authors participated in reviewing and editing the manuscript and approved the final version.

## Conflicts of interest

The authors declare no conflict of interest.

## Supplementary Material

NA-008-D6NA00219F-s001

## Data Availability

The data supporting the findings of this work are available within the article. Supplementary information (SI): synthesis of W_18_O_49_-NWs and pristine WS_2_-NTs; preparation of ampoule for synthesizing Re-doped WS_2_-NTs (Fig. S1); FESEM, TEM and HRTEM images of W_18_O_49_-NWs (Fig. S2); structural evolution of pristine WS_2_-NTs (Fig. S3); temperature dependent study of Re-doped WS_2_-NTs (Fig. S4); reaction time dependent synthesis of Re-doped WS_2_-NTs (Fig. S5); effect of ReO_3_ precursor concentration on the morphology and composition of Re-doped WS_2_-NTs (Fig. S6); EDS of Re-doped WS_2_-NTs (Fig. S7); fitting graphs for determining resistance, and carrier density (Fig. S8). See DOI: https://doi.org/10.1039/d6na00219f.
