# Supplementary material for: Synthesis and electrical characterization of rhenium-doped WS2 nanotubes
Source: Nanoscale Adv. 2026 May 29;8(14):3975–80. doi: 10.1039/d6na00219f (PMC13237786; doi:10.1039/d6na00219f)
Supplement: NA-008-D6NA00219F-s001 [file NA-008-D6NA00219F-s001.pdf]

Supplementary Information (SI) for Nanoscale Advances.
This journal is © The Royal Society of Chemistry 2026

Supporting information

Synthesis and Electrical Characterization of Rhenium-Doped WS_2 Nanotubes

Abdul Ahad,[a,b] Saitou Shigeki,[a] Ryuji Higashinaka,[a] Masaharu Kikuchi,[a] Satoshi Kusaba,[a]

Zheng Liu,[d] Yasushi Hirose,[c] and Kazuhiro Yanagi,[a]

[a]Department of Physics, Tokyo Metropolitan University, Hachioji, Tokyo 192-0397, Japan

[b]Department of Physics, Comilla University, Cumilla-3506, Bangladesh

[c]Department of Chemistry, Tokyo Metropolitan University, Hachioji, Tokyo 192-0397, Japan

[d]Muti-Material Research Institute, National Institute of Advanced Industrial Science and Technology (AIST), Nagoya 463-8560, Japan

E-mail: yanagi-kazuhiro@tmu.ac.jp

1. Synthesis of $W_{18}O_{49}$-NWs and pristine WS_2-NTs

The CVD-grown $W_{18}O_{49}$-NWs were synthesized on the c-plane sapphire substrate within a three-zone electric furnace following our previous study,[1] the only difference was the temperature. Here, we used upstream temperature 880 °C and downstream temperature 680 °C. In the case of pristine WS_2-NTs synthesis, we also used our previous system and conditions[1] excepting conversion temperature. For proper sulfurization, we used NWs' temperature 760 °C and sublimation temperature of sulfur lump 250 °C.

1.1 Preparation of ampoule for synthesizing Re-doped WS_2-NTs

To prepare a 25 cm long ampoule, we started with a 100 cm long quartz tube. The tube was divided in half using an N_2/O_2 gas burner, resulting in two 50 cm long sections, each with one end sealed. We placed 12 mg of ReO_3 as the precursor of Re atoms and 15 mg of I_2 as the carrier into one end of the tube (designated as point A). To prevent the substrate from dropping into point A, we created a neck approximately 15-16 cm away from the ReO_3 and I_2.

Next, we inserted the substrate containing pristine WS_2-NTs into the tube at point B. After inserting the substrate, we formed another neck at point C, approximately 9–10 cm from point B. This neck was made particularly deep because it would later be sealed. The tube was then connected to a rotary pump and a diffusion pump, achieving a vacuum of approximately 5×10^{-5}–10^{-4} Pa. Once the vacuum was established, the tube was sealed, completing the preparation of the 25 cm long ampoule. The same procedure was followed to prepare another ampoule using the remaining 50 cm section.

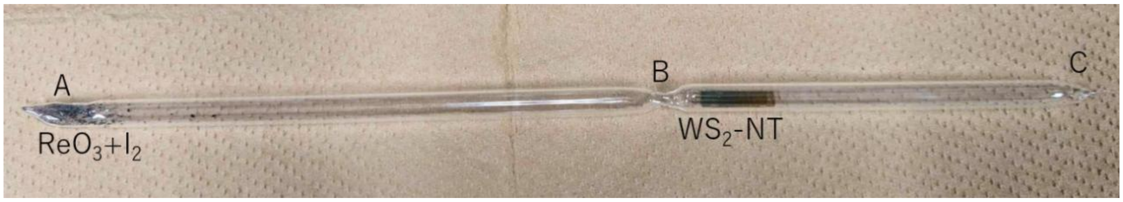

Fig. S1: A photo of the ampule contains ReO_3, I_2 reagent, and a sapphire substrate on which pristine WS_2-NTs were synthesized.

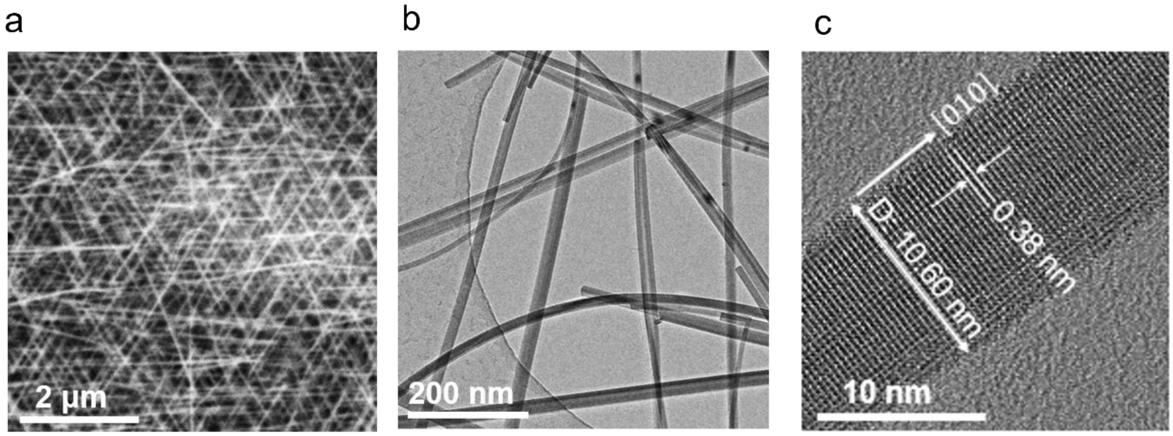

Fig. S2 CVD-grown $W_{18}O_{49}$-NWs on the sapphire substrate. (a) SEM, and (b) low magnified TEM image shows the flake free morphology of the samples. (c) HRTEM image expresses the crystal structure and growth direction of the individual NWs. Our studied NWs were confirmed as single crystalline, exhibiting a lattice spacing of 0.38 nm along the [010] growth direction, which corresponds to the monoclinic non-stochiometric phase of $W_{18}O_{49}$.[2,3]

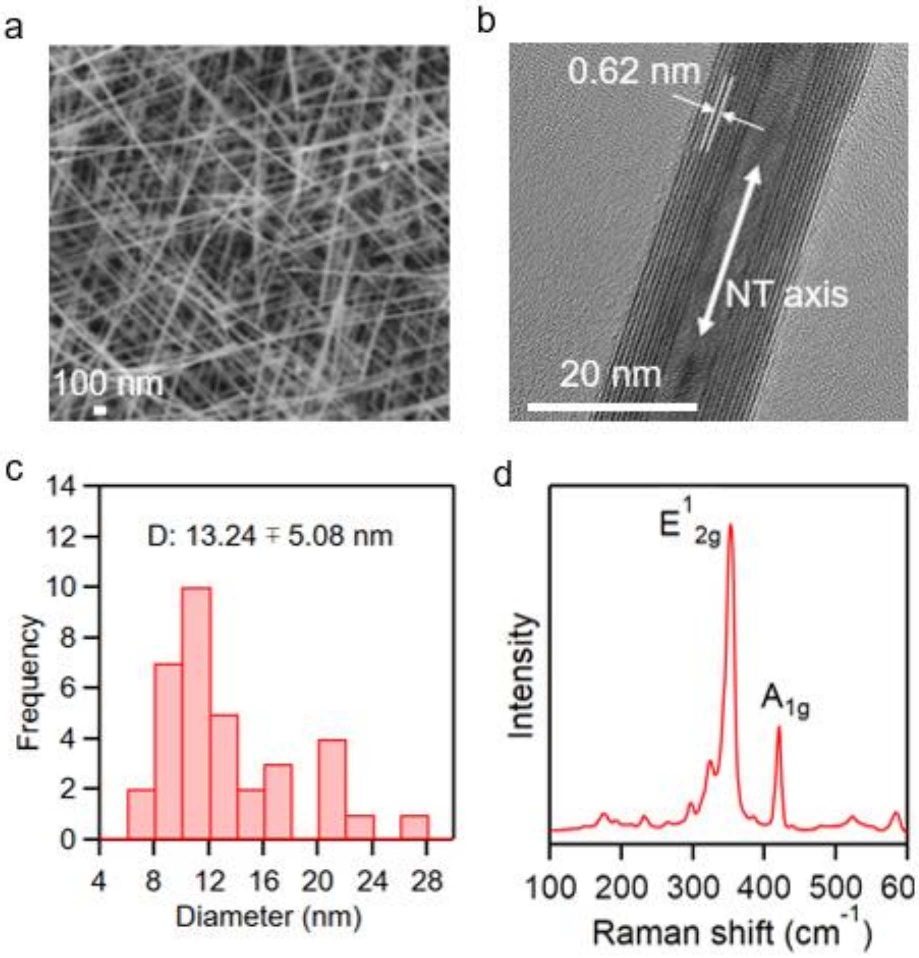

Fig. S3 Structural evolution of pristine WS_2-NTs. (a) SEM image, (b) HRTEM image indicating the NT axis direction with a white arrow, (c) Diameter distribution histogram of the NTs with standard deviation, where 35 NTs were used in this analysis, and (d) Raman shift of pristine WS_2-NTs. In-plane and out-of-plane Raman mode at 354 and 419 cm^{-1}.

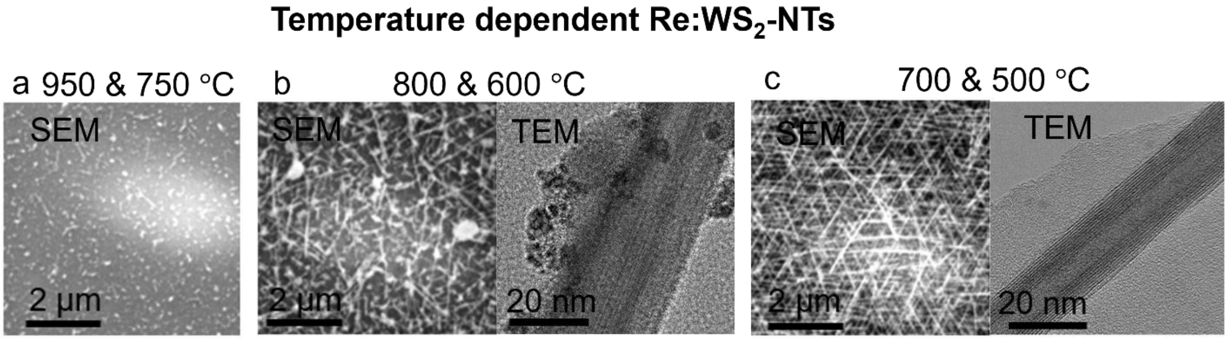

Fig. S4 (a) Due to high synthesis temperature, the majority of the NTs decomposed, resulting in the formation of particle-like structures. Owing to the loss of tubular morphology, further structural characterizations were not carried out for these samples. Subsequently, the synthesis temperatures were reduced, and SEM imaging (b) revealed the presence of tubular structures. HRTEM image showed partially broken NTs with poor crystallinity. EDS analysis confirmed the presence of Re at approximately 22 at.%, which was significantly higher than anticipated. Additionally, the W:S atomic ratio 9:2 (W: 46 at.%, S: 10 at.%), indicating sulfur deficiency and suggesting that a further reduction in synthesis temperature is necessary to improve stoichiometry and structural integrity. (c) When a temperature gradient of 200 °C was applied, with the high-temperature zone at 700 °C and the low-temperature zone at 500 °C, the formation of well-defined nanotubular morphology and crystalline structure was observed, as expected.

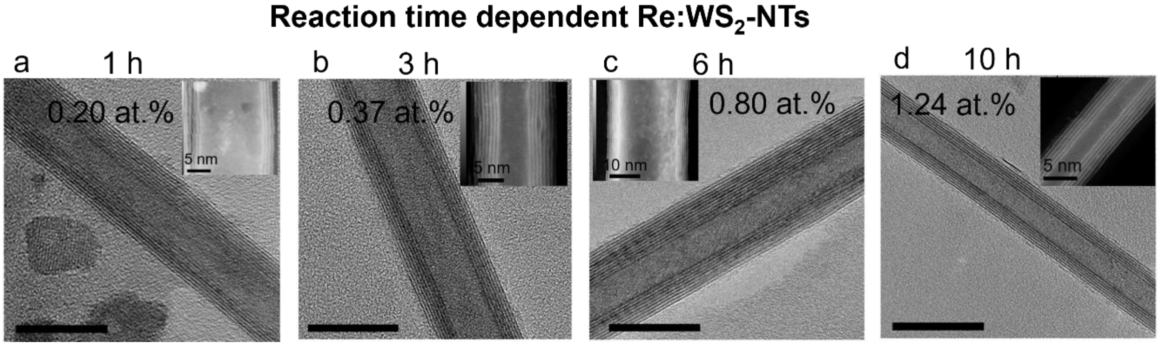

Fig. S5 Re-doped WS_2-NTs were synthesized under varying reaction times. The scale bar is 20 nm. At shorter durations (a), particle-like structures were observed alongside the NTs. In contrast, at longer reaction times (b-d), well-formed nanotubular structures were obtained. Considering improved morphological uniformity and higher Re incorporation, a reaction time of 10 hours was selected for further studies.

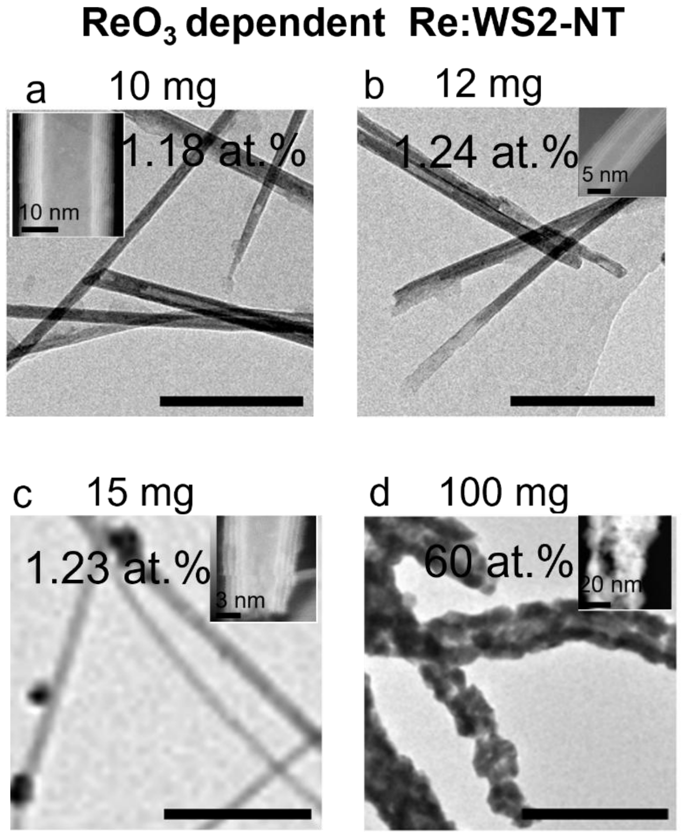

Fig. S6 A series of experiments were conducted to investigate the effect of ReO_3 precursor concentration on the morphology and composition of Re-doped WS_2-NTs. The scale bar in all images is 200 nm. Successful synthesis of Re-doped WS_2-NTs was achieved using 10-12 mg of ReO_3 powder (a-b), with 12 mg yielding more uniform and well-formed NTs. However, when the ReO_3 amount was increased to 15 mg (c), a significant number of particle-like structures appeared on the sample surface, indicating morphological degradation. To further examine the effect of excessive Re, the ReO_3 quantity was drastically increased to 100 mg (d). Elemental analysis revealed an Re concentration of approximately 60 at.% and a W content of only 4 at.%, along with 33 at.% oxygen. These results suggest that under such conditions, the WS_2-NTs were nearly converted into ReO_3-based wire-like structures.

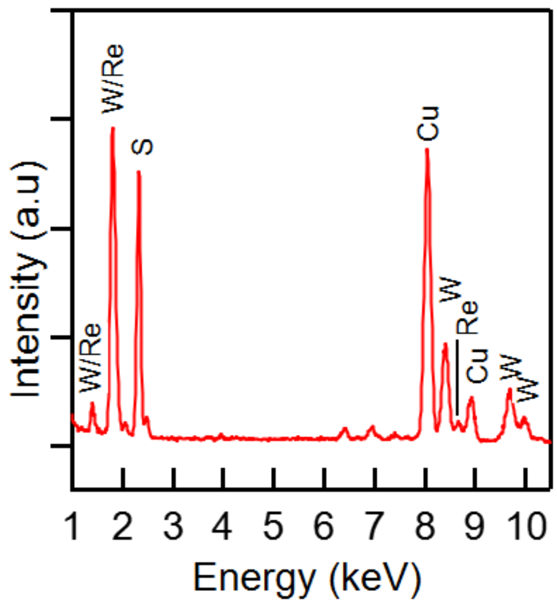

Fig. S7 EDS spectra of Re-doped WS_2-NTs. Due to small energy differences, the X-ray emission line of W and Re overlaps at M-line region.

2. Analysis procedure of I-V characteristics using Metal-Semiconductor-Metal (M-S-M) model.

We calculated Schottky barriers heights using metal-semiconductor-metal (M-S-M) model.[4] We assume the Schottky barrier 1, WS_2-NT (NT), and Schottky barrier 2. When the channel voltage V is applied between the two metal electrodes, the total voltage is denoted as

$$V = V_1 + V_{NT} + V_2 \tag{1}$$

Here V_1, and V_{NT} and V_2 are voltage drops at the barrier 1, NT and the barrier 2, respectively. In the two Schottky barriers, one is the forward biased and the other is the reverse biased. According to the thermionic emission theory, the current density is written as

$$J(V, \phi_b) = A^* T^2 \exp\left(-\frac{\phi_b}{kT}\right)\left(\exp\left(\frac{qV}{kT}\right) - 1\right) \tag{2}$$

Here $A^* = 4\pi m^* q k^2/h^3$ is the Richardson constant of the semiconductor, m^* is effective mass, ϕ_b is the effective Schottky barrier height, k is the Boltzmann constant, q is the magnitude of the electronic charge, T is the absolute temperature. Here the forward biased current ($V > 0$) is taken as positive, and the reverse biased ($V < 0$) current is taken as negative. In the nano-M-S-M structure, tunneling through the reverse-biased Schottky barrier becomes significant.[4] Thus, the current density of the one reverse biased (for consistency, here $V < 0$ and the sign of the current is taken as negative) is,

$$J(|V|, \phi_b) = -J_{ST}(|V|, \phi_b) \exp\left[|V|\left(\frac{q}{kT} - \frac{1}{E_0}\right)\right] \tag{3}$$

Here

$J_{ST}(|V|, \phi_b) = \frac{A^* T(\pi q E_{00})^{1/2}}{k} \exp\left(-\frac{\phi_b}{kT}\right)\left\{q(|V| - \zeta) + \frac{\phi_b}{\cosh^2(qE_{00}/kT)}\right\}^{1/2}$ with ζ is the distance between the Fermi energy to the bottom of the conduction band, and $E_0 = E_{00}\coth\left(\frac{qE_{00}}{kT}\right)$, $E_{00} = \frac{\hbar}{2}\left[\frac{N_d}{m^* \varepsilon_s \varepsilon_0}\right]^{\frac{1}{2}}$, N_d is the doner density, ε_s is the relative permittivity of the nanotube, and ε_0 is the permittivity of free speace.

The current I flowing through the three components is the same, and thus

$$I = S_1 J_1(V, \phi_b) = V_{NT}/R_{NT} = S_2 J_2(V, \phi_b) \qquad (4)$$

Here S_1 and S_1 are the contact areas of the Schottky barrier 1 and 2, and R_{NT} is the resistance of NT. Here we ignored the shunt resistances of the barrier 1 and 2.

Here we used the effective Richardson constant of WS_2-NT = 5.7×10^5 Am^2K^{-2} with $m^* = 0.4 m_0$,[5] and ε_s is 1.20.[5]

According to the suggested approach to determine the parameters,[4] we determined the parameters as follows. First, the R_{NT} is determined by differentiating the I-V curve at around 10 V (Fig. 4(c-d)), then the determined the R_{NT} is used for the simulation as the initial parameter, respectively. After determining the R_{NT}, the I-V curve is replotted in logarithmic scale (Fig. S8(a-b)), and from the In(I) versus V at intermediate bias, then the E_0 is determined from the slope of the linear component since the slope is given by

$$slope = \frac{q}{kT} - \frac{1}{E_0}$$

and then N_d is determined. The $E_0 = 0.0261$ eV for pristine and $E_0 = 0.0263$ eV Re-doped WS_2-NTs, respectively, and thus $N_d = 3.0 \times 10^{17}$ cm^{-3} for pristine and $N_d = 4.9 \times 10^{17} cm^{-3}$ for Re-doped WS_2-NT. The ϕ_b is determined numerically from Eqs (1), (2), (3) and (4) through the fitting on the I-V characteristics. The fitted results are shown in Figures 4 (c) and (d) in the main manuscript. Finally, the R_{NT} for pristine WS_2-NT is determined to be 4.4×10^{11} Ω, and that of Re-doped NT is 2.6×10^9 Ω. According to the NT diameters (19.4 nm for pristine and 11.3 nm for Re-doped which are determined by the AFM measurements) and channel length (3.0 μm), the electrical conductivity is determined to be $2.3 \times 10^{-2}\ Sm^{-1}$ for pristine WS_2-NT, and $1.1 \times 10\ Sm^{-1}$ for Re-doped NT. In addition, the Schottky barrier $\phi_b = 0.75\ eV$ is estimated for

pristine, and $\phi_b = 0.67$ eV for Re-doped, and the $\zeta = 0.72\ eV$ for preistine, $\zeta = 0.65\ eV$ for Re-doped WS_2-NT.

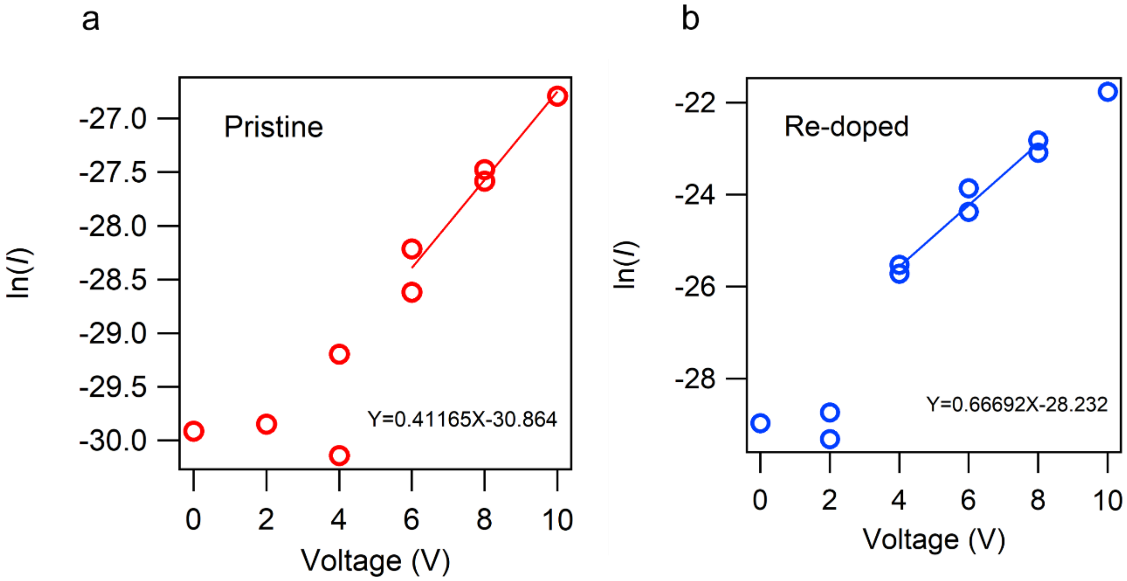

Fig. S8 The ln(I)–V graphs of pristine (a) and Re-doped (b) WS_2-NTs. From the slope, we determined the characteristic energy E_0, and subsequently the carrier density of the samples.

References

1. A. Ahad, Y. Yomogida, M.A. Rahman, A. Ihara, Y. Miyata, Y. Hirose, K. Shinokita, K. Matsuda, K.; Z. Liu and K. Yanagi, K. *Nano Lett*., 2024, **24**, 14286-14292.
2. K. Hong, M. Xie, R. Hu and H. Wu, *Appl. Phys. Lett.*, 2007, **90**, 173121.
3. H. Zhang, Y. Wang, S. Zuo, W. Zhou, J. Zhang and X. W.D. Lou, *J. Am. Chem. Soc.,* 2021, **143**, 2173-2177.
4. Z. Zhang, K. Yao, Y. Liu, C. Jin, X. Liang, Q. Chen and L. M. Peng, *Adv. Funct. Mater.,* 2007*,* **17**, 2478-2489.
5. A. Sengupta, and S. Mahapatra, *J. Appl. Phys.,* 2013, **113,** 194502.
